# Effects of Home Nursing and Rehabilitation on Daily Life and Outgoing Activities in a Patient With Interstitial Pneumonia Supported by a High-Flow Nasal Cannula: A Case Report

**DOI:** 10.7759/cureus.72691

**Published:** 2024-10-30

**Authors:** Toshiki Azuma, Shinichi Onozawa, Yukari Miyamoto, Nobuyuki Katayama

**Affiliations:** 1 Rehabilitation Medicine, Yawata Medical Center, Komatsu, JPN; 2 Visiting Nurse Station, Rehabilitation Care Rojo, Komatsu, JPN; 3 Rehabilitation, Asanogawa General Hospital, Kanazawa, JPN; 4 Respiratory Medicine, Yawata Medical Center, Komatsu, JPN

**Keywords:** chronic obstructive pulmonary disease, high-flow nasal cannula, home respiratory rehabilitation, interstitial pneumonia, respiratory failure

## Abstract

A high-flow nasal cannula is used as the primary therapy for patients with respiratory failure. Its effectiveness, however, is contingent upon proper patient monitoring by trained healthcare professionals. The case report highlights the benefits of home nursing and rehabilitation in an 81-year-old woman with rheumatoid-related interstitial pneumonia (complicated by drug-induced interstitial pneumonia) who developed respiratory failure. She lived at home with her family while undergoing high-flow therapy. At the outset of the home visit care, she was bedridden, had an indwelling urinary catheter, ate with assistance, and had to wipe her entire body because she could not bathe. Five months after receiving home visit care, she was able to use a portable toilet. However, she was unable to walk because of dyspnea and muscle weakness. She then went through five months of home visit rehabilitation before being able to move around her home with a walker. In addition, she was able to ride out in the back seat of her husband's car. Her quality of life improved as her daily activities improved, and she was able to participate in leisure activities. This experience suggests that home visit rehabilitation may be beneficial in promoting outdoor activities in patients with interstitial pneumonia caused by severe respiratory failure.

## Introduction

Pulmonary fibrosis is a common type of interstitial pneumonia (IP) that can lead to respiratory failure due to extensive fibrosis and cellulite in the interstitium of the lungs [1]. Advanced pulmonary fibrosis causes dyspnea on exertion, as well as hypoxemia, which impairs daily life [2]. IP can cause recurrent acute exacerbations, which are associated with a low chance of survival [2]. Even if patients with acute exacerbations of IP can return home following acute treatment, their quality of life (QOL) is frequently compromised due to limited activities of daily living (ADL) and dyspnea [3]. Thus, they require an additional management approach to improve their daily functions. Recently, the efficacy of a high-flow nasal cannula (HFNC) has been found in patients with acute exacerbations of respiratory failure, and it became available for home use in Japan in April 2022 [4-6]. However, there are few studies demonstrating the efficacy of home visit rehabilitation for patients who use HFNC at home. Generally, it has been reported that home visit rehabilitation for patients with respiratory failure improves their QOL [7]. We hypothesized that home rehabilitation would improve the QOL for patients with respiratory failure using HFNCs. In this case, we performed home respiratory rehabilitation on a patient who had IP due to respiratory failure and was expected to receive care at home.

## Case presentation

**Figure 1:**
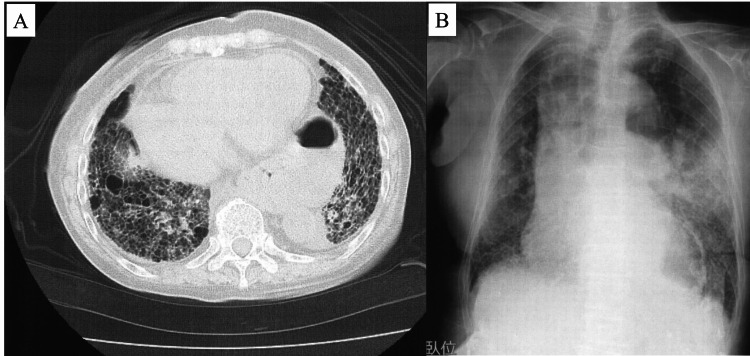
X day (admission) Chest CT results showed an exacerbation of pulmonary interstitial shadows, mainly frosted shadows (A). The X-ray images showed frosted shadows in the middle and lower lobes (B).

An 81-year-old woman complained of difficulty in walking indoors and outdoors, as well as being unable to go out in her husband’s car. The patient’s height, weight, and body mass index were 148.0 cm, 42.0 kg, and 19.2 kg/m², respectively. She was diagnosed with rheumatoid arthritis 30 years ago and has been treated accordingly. Seven years before her first visit to our hospital, she developed IP, was diagnosed with pulmonary hypertension and chronic obstructive pulmonary disease (COPD), and was prescribed sildenafil. She eventually developed respiratory failure and was treated with home oxygen therapy at 2.0 L/min. In addition, she was on tacrolimus and abatacept for rheumatoid arthritis. On X - 60 days, she was given tiotropium bromide hydrate and olodaterol hydrochloride for dyspnea. Whereas on X - 45 days, she had a cough and dyspnea, and her SpO₂ fell below 70% upon inhalation of 2.0 L/min oxygen, necessitating admission to an emergency clinic. After her chest CT revealed an acute exacerbation of interstitial pneumonia, she was admitted to the hospital and treated with steroid pulse therapy. The respiratory condition improved, and the steroid was tapered off. X - 30 days, the patient’s oxygen uptake returned to pre-exacerbation levels, and she was released from the hospital. X - 20 days, after the prednisolone dose was reduced to 7.5 mg, abatacept was administered as an outpatient. X days, dyspnea developed, and the patient was admitted to the emergency room with oxygen therapy at 5.0 L/min and SpO₂ at 55% (Figure 1, Table 1).

**Table 1:** Blood collection data WBC: White Blood Cell Count, Hb: Hemoglobin, Alb: Albumin, BUN: Blood Urea Nitrogen, Cr: Creatinine, Na: Sodium, K: Potassium, CRP: C-Reactive Protein, KL6: Sialylated Carbohydrate Antigen, BNP: Brain Natriuretic Hormone, PH: Potential Hydrogen, PaCO2: Partial Pressure of Arterial Carbon Dioxide, PaO_2_: Partial Pressure of Arterial Oxygen, HCO_3_⁻: Hydrogencarbonate, BE: Base Excess At the time of admission, the patient had type I and type II respiratory failure and worsening heart failure. At X + 300 days, the type I respiratory failure had improved slightly, and the heart failure had improved.

Day	X days	X＋300 days	
Valuable	Admission	Post rehabilitation	Normal range
WBC (10³/㎕)	9300	9700	4000-8000
Hb (g/dL)	10.9	11.8	13.5-17.6
Alb (g/dL)	2.6	3.1	3.4-5.0
BUN (mg/dL)	21.3	29.9	8-22
Cr (mg/dL)	0.48	0.91	0.65-1.07
Na (mEp/L)	144	145	138-146
K (mEp/L)	3.6	4.3	3.5-5.0
CRP (mg/dL)	2.48	0.35	0-0.30
KL6 (U/mL)	504	300	<500
BNP (pg/mL)	345	50.5	<18.4
HbA1c (%)	5.2	5.4	<6.5
PH (mol)	7.4	7.4	7.4
PaCO_2_ (mmHg)	45.3	51.8	35-45
PaO_2_ (mmHg)	39.4	271	80-100
HCO_3_^⁻^(mol/L)	25.3	32.8	24
BE (mol/L)	-0.5	6.8	-2-2

High-dose steroid therapy (methylprednisolone 1 mg/kg) was found to be ineffective for acute exacerbation. Consequently, HFNC (30 L, FiO_2_ 0.7) was prescribed concurrently, but the hypoxemia did not improve. The patient and her family were strongly advised to seek palliative care at home using the HFNC, and she was discharged home on X + 30 days after balloon catheter placement. The patient, however, required complete assistance with ADL. After being discharged, she received home care from her two daughters (in their 50s) from other prefectures, who alternated visits to her home due to the need for a balloon catheter and full assistance with ADL. After discharge, visiting nurses started providing body cleaning and assistance with eating while sitting. During home visits, nurses observed and evaluated whether or not there was any nasal mucosal damage caused by HFNC. As she was unable to go out, she received visiting medical care from a doctor. After consulting with the visiting doctor and nurse, they set aside an hour for her to leave the HFNC. During the X + 150 days, her ADL gradually increased. While using HFNC (25 L, FiO_2_ 0.4), she was able to defecate in a portable toilet placed next to her bed under the supervision of a caregiver and watch TV while sitting. She wanted to broaden her daily living options, so home-visit rehabilitation began (X + 150 days).

She was on tacrolimus tablets, vonoprazan tablets, mosapride citrate tablets, sildenafil citrate, furosemide tablets, prednisone tablets, ferric citrate sodium tablets, pantethine tablets, and thiotropium bromide hydrate.

(Rehabilitation Evaluation: X + 150 days)

Overall picture at the start of home visit rehabilitation: HFNC (25 L FiO_2_ 0.4) use prevented dyspnea and hypoxemia while sitting up, and the patient could communicate effectively. When walking with 7.0 L/min of home oxygen therapy, SPO_2_ was 80%, making walking difficult. The patient’s mental status was more limited in ADLs at the start than at X - 30 days, and both the patient and her family were very anxious. The patient also expressed dissatisfaction with her long sitting time. A physical examination revealed no limitations in lower limb and trunk joint range of motion, sensory disturbance, or abnormal reflexes. However, she had difficulty walking due to dyspnea and decreased overall endurance caused by weakness of the lower limb muscles. This patient had rheumatoid arthritis, but the walking disorder was not due to joint pain or limited range of motion. The cause of the walking disorder was decreased muscle endurance and cardiopulmonary endurance, which were caused by low activity due to IP and heart failure (Tables 2-3).

**Table 2:** Physical function data SPPB: Short Physical Performance Battery, MRC score: Medical Research Council Score; SGRQ: St. George's Respiratory Questionnaire, BI: Barthel Index, LSA: Life Space Assesment A higher total score indicates better SPPB, BI, and LSA; a lower total score indicates better SGRQ and MRC scores.

Day	X＋150 days	X＋300 days	
Valuable	Start rehabilitation	Post rehabilitation	Total possible score range
Knee extension strength (Rt/Lt, N/kg)	18/17	40/35	0-100
Grip strength (Rt/Lt, kg)	12/10	16/15	0-100
SPPB (point)	0	10	0-12
Six-minute walk test (m)	3 (Walker)	140 (Walker), 60m (Cane)	0-700
MRC grade (scale)	5	4	0-5
SGRQ (Symptom/Activities/Impact/total, point)	69/95/100/88	44/57/76/59	0-100
BI (point)	40	80	0-100
LSA (point)	2	36	0-120

**Table 3:** Rehabilitation program

Rehabilitation program	Detail
Walking practice and leading home exercise	Walking exercises were performed with attention to SPO₂ not falling below 90% and dyspnea not exceeding 15 on the Borg scale. Patients and family members were instructed to use an oximeter so that they could perform walking exercises on their own. 1-10 sets of walking exercises were performed depending on hypoxemia and dyspnea. 3 m was started and up to 140 m was performed at X + 300 days.
ADL practice	ADL practice was conducted in stages. They were asked to wash their face and brush their teeth in a standing position, walk 3 m to a portable toilet, walk 5 m to the kitchen and eat, walk 7 m to the bathroom, walk 10 m to the toilet, and walk 20 m to the car outside the house. With improvement in walking ability, step climbing exercises were performed at the front door and in front of the house. Management of hypoxemia and dyspnea was similar to the walking exercises.
Go out practice	They practiced walking, climbing up and down steps, and getting in and out of the back seat of a car. These practices alone did not allow him to go out in a car. She practiced with her daughter and husband administering oxygen cylinders; each 7.0L oxygen cylinder was durable for 50 minutes and needed to be replaced as needed.
Patients education	We explained the risk of falls associated with walking exercises and the risk of exacerbation of respiratory and cardiac failure associated with hypoxemia. We instructed my daughter and her husband to take vital measurements on paper. She instructed them to perform activities at home, noting increased dyspnea at rest and no sympathetic hyperactivity (increased resting blood pressure and pulse rate).

(Rehabilitation evaluation: X + 300 days)

**Figure 2:**
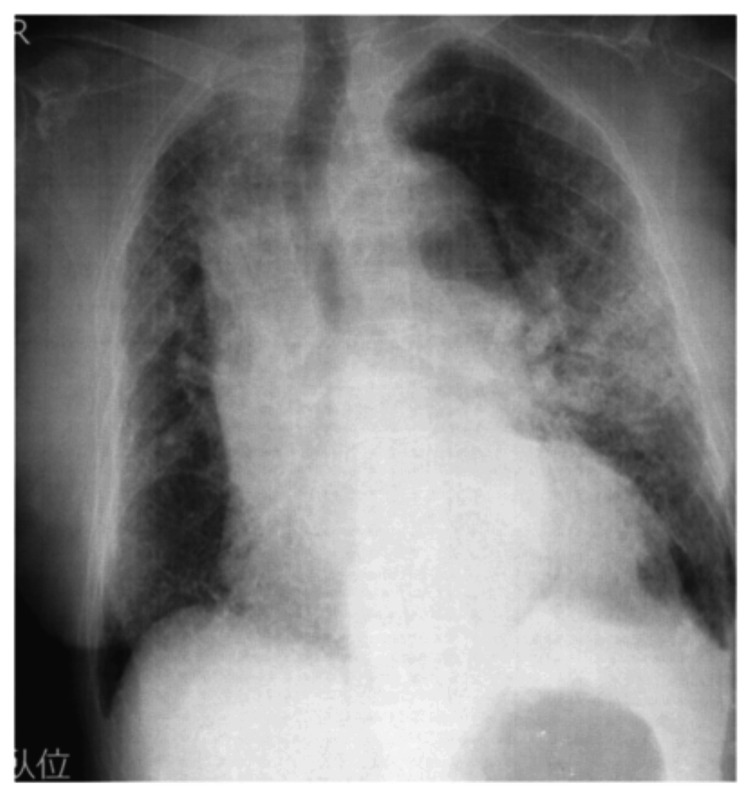
X＋ 300 days (post rehabilitation) Compared to X day, the bilateral lower lung interstitial shadows have improved.

In this case, the patient and family were unable how to go out in the car without worsening the patient's breathing condition at X + 250 days, even after gait acquiring and improving sitting endurance. This was due to the patient’s and family’s anxiety, as well as inadequate oxygen cylinder management. Therefore, we explained the patient’s current physical function test results, as well as the patient’s current oxygen cylinder management. The HFNC was used for an hour in the morning and eight hours at night. During the day, the HFNC was removed, and the patient was able to sit for three to four hours while using home oxygen (7.0 L/min) therapy. The patient’s knee extension muscle strength and dyspnea improved, and she was able to walk with a walker, use the restroom, and shower at home. Using a walker, the patient was able to walk continuously for 140 m without experiencing dyspnea or hypoxemia. Additionally, she could walk up and down the front door steps and leave for up to three hours while sitting in the back seat of a car. She also reported an improved QOL (Tables 2-3). There was no respiratory or cardiac failure post rehabilitation (Figure 2, Table 1).

## Discussion

Home nursing and rehabilitation demonstrated significant improvement in daily activities and QOL in an 81-year-old woman with rheumatoid-associated IP. The patient was initially bedridden and dependent, but she progressed to using a walker and participating in leisure activities, demonstrating the efficacy of home visit rehabilitation. Her ability to perform daily activities independently and engage in hobbies suggests that home nursing and rehabilitation significantly improve the QOL for patients with severe respiratory failure. While previous studies have reported the effectiveness of home morphine treatment for patients with severe respiratory failure using HFNC [8], there have been no reports on supporting patients with severe respiratory failure using HFNC for outdoor activities, making this approach novel. The patient had dyspnea and decreased general endurance due to hypoxemia. Her inability to walk and limited mobility in ADLs increased anxiety for the patient and her family, resulting in a lower QOL. Home nursing care gradually increased ADLs in collaboration with the patient’s primary care physician, allowing her to maintain a sitting position, eat, and use a portable toilet. During home rehabilitation, walking and aerobic exercises were performed using home oxygen, which improved the patient’s overall endurance. Consequently, she was able to move around the house with a walker, ascend and descend steps, and sit in a car for extended periods, allowing her to drive. These expanded activities reduced anxiety for the patient and her family while improving QOL.

The patient and family were very concerned about the ambulation gap between X - 30 days and X + 150 days. Using a problem-solving technique, we determined which daily life tasks had the highest cardiopulmonary burden and shared goals [9]. The daily living tasks were graded in the following order: portable toilet elimination using HFNC as the easiest, prolonged sitting time using home oxygen, dressing activities, walking with a walker in the hallway, ascending and descending steps at the entrance, and driving for extended periods. By organizing these tasks, the patient and family were able to better understand the tasks required for the drive and participate in rehabilitation with less anxiety. There were several reports that HFNC improves dyspnea and quality of life after discharge [4,10]. The initial rehabilitation evaluation of this patient was X + 150 days, which was a lot of time after the use of HFNC. The improvement of 29.0 points in St. George’s Respiratory Questionnaire (SGRQ) before and after rehabilitation intervention is a greater improvement than the 9.7 points in previous studies. This suggests that home-visit rehabilitation had a positive impact on mental health.

A gradual range-of-life extension was performed to address the patient’s decreased general endurance, which impeded gaining ambulation. The initial goal was to increase sitting time during home oxygenation while partially discontinuing HFNC. It had a risk of increased carbon dioxide retention and dyspnea caused by hypoxemia [4-6]. In addition, the patient had chronic heart failure, which exacerbated dyspnea. To address these concerns, patient and family education was provided to increase awareness of heart failure exacerbation and dyspnea. Gradual expansion of the patient’s living arrangements proved effective in improving physical function and increasing ADLs. The patient’s living range of motion was altered by extended sitting time on home oxygen, walking with a walker to a portable toilet, walking with a walker in the hallway, and gaining and regaining the ability to ascend and descend stairs. The improvement in these activities was attributed to an improvement in physical function: at the end of X + 300 days of home rehabilitation, the patient was able to stand continuously for more than five minutes, had high muscle strength values, and improved endurance, with isometric knee extension muscle strength (average body weight ratio: 35%-40%). The patient also received a Short Physical Performance Battery score of 10, which measures balance ability in a standing position. According to a previous study, the cutoff values for quadriceps strength for independent-level walking with a walker or cane were approximately 17% and 43%, respectively [11]. Home-visit rehabilitation improves balance and walking ability while lowering the risk of falling in patients with respiratory diseases. The rehabilitation effect on IP has been reported as an 81-meter improvement in 6MWT after 12 weeks of exercise therapy as a report on the QOL, and a 9.7-point improvement was obtained in a previous study [12-16]. The present case showed a large improvement in general endurance and QOL compared to these reports. This may be due to the possibility that the present patient had concurrent disuse syndrome after drug-induced IP.

In this case, the patient was unable to learn driving activities at X + 250 days, even after gait acquiring and improving sitting endurance. This was due to the patient’s and family’s anxiety, as well as inadequate oxygen cylinder management. Therefore, we explained the patient’s current physical function test results, as well as the patient’s current oxygen cylinder management. The patient was able to sit for approximately five hours and walk more than 100 meters continuously with 7.0 L/min of home oxygen. However, the 7.0 L/min had a limited lifespan, lasting only 50 minutes per cylinder, and there were anxieties about the management of oxygen cylinders by family members. We repeatedly practiced checking the oxygen cylinders’ remaining capacity and efficiently changing them. The family’s anxiety level gradually decreased. As a result, the patient completed a three-hour ride in a car driven by her husband. This intervention greatly enhanced the patient’s QOL.

## Conclusions

The use of HFNC at home has not been approved for patients with IP. In this case, the patient had COPD, which allowed HFNC to be used at home after the exacerbation of IP. The findings of this case may help expand the indications for HFNC. A patient with ADL limitations caused by repeated acute exacerbations of IP received home nursing care and rehabilitation. The patient could perform ADLs with a walker and ride in the back seat of a car for a go-out. The expansion of the patient’s life sphere resulted in an improvement in QOL. Our findings indicate that home visit rehabilitation can effectively improve ambulation and leisure activities in patients with severe IP using HFNC.
